# The role of AI-driven decision support in immunotherapy for predicting pathological complete response in triple-negative breast cancer

**DOI:** 10.3389/fonc.2026.1798015

**Published:** 2026-04-13

**Authors:** Di Lu, Xihui Tu, Yaling Zeng, Huixi Zhong, Tengfei Xing, Yuying Wang, Rui Wang, Purong Zhang

**Affiliations:** 1 School of Medicine, University of Electronic Science and Technology of China, Chengdu, Sichuan, China; 2 Department of Breast, Sichuan Clinical Research Center for Cancer, Sichuan Cancer Hospital & Institute, Sichuan Cancer Center, University of Electronic Science and Technology of China, Chengdu, Sichuan, China; 3 College of Clinical Medicine, North Sichuan Medical College, Nanchong, Sichuan, China; 4 Department of Health Management Center, Qionglai Medical Center Hospital, Qionlai, Sichuan, China; 5 Sichuan Mianyang 404 Hospital, Mianyang, Sichuan, China

**Keywords:** immunotherapy, machine learning, neoadjuvant chemotherapy, pathological complete response, pembrolizumab, triple-negative breast cancer

## Abstract

**Background:**

Neoadjuvant pembrolizumab combined with chemotherapy has become a standard treatment strategy for early-stage triple-negative breast cancer (TNBC). However, real-world evidence regarding its effectiveness and models for predicting pathological complete response (pCR) remain limited.

**Methods:**

We retrospectively collected data from 248 patients with TNBC who received neoadjuvant chemotherapy (NACT) with or without pembrolizumab followed by surgery between May 2023 and December 2025. Patients were categorized into two groups: the pembrolizumab-chemotherapy group (n = 40) and the chemotherapy-alone group (n = 208). Propensity score matching (PSM) was applied to balance baseline characteristics. Machine learning models were developed using routinely available clinical and pathological variables to predict pCR, and model interpretability was evaluated using SHapley Additive exPlanations (SHAP).

**Results:**

Both before and after PSM, the pembrolizumab–chemotherapy group achieved a higher pCR rate than the chemotherapy-alone group (before: 50.0% vs. 30.8%, p = 0.030; after: 47.2% vs. 26.5%, p = 0.037). LASSO regression selected six variables associated with pCR, including pembrolizumab, age, sum of diameters of target lesions (SLD), Ki67, N stage, and clinical stage. In the training cohort, these six variables were used to develop eight machine learning models to predict pCR. In the validation cohort, the MLP model achieved the highest receiver operating characteristic–area under the curve (ROC-AUC) of 0.71. The calibration curve and decision curve analysis (DCA) further indicated good calibration and clinical utility of the MLP model.

**Conclusions:**

Neoadjuvant pembrolizumab combined with chemotherapy further improved pCR rates in TNBC, and the MLP-based model demonstrated good performance for predicting pCR.

## Introduction

Triple-negative breast cancer (TNBC) is the breast cancer subtype associated with the poorest prognosis and is characterized by high aggressiveness and marked biological heterogeneity (1–3). According to data from the American Cancer Society (2014–2020), the average 5-year survival rate for patients with TNBC is approximately 73%–81%, which is 11–17 percentage points lower than that for patients with hormone receptor–positive/human epidermal growth factor receptor 2–negative (HR+/HER2−) breast cancer (4). Neoadjuvant chemotherapy (NACT) followed by surgery remains the cornerstone of treatment for TNBC (5, 6). Pathological complete response (pCR) after NACT has been widely accepted as a critical indicator of treatment efficacy and a reliable surrogate endpoint for long-term survival outcomes (7–9).

In recent years, immunotherapy has substantially advanced the neoadjuvant treatment of TNBC. The KEYNOTE-522 trial demonstrated that the addition of pembrolizumab to NACT significantly increased the pCR rate to 64.8%, compared with 51.2% achieved with NACT alone (p < 0.001) (10). Moreover, Schmid et al. confirmed that the 3-year event-free survival (EFS) was superior in early-stage TNBC patients treated with pembrolizumab–chemotherapy compared with placebo–chemotherapy (84.5% vs. 76.8% p < 0.001) (11).

Although pembrolizumab has demonstrated favorable efficacy in TNBC, real-world evidence remains limited. Several independent retrospective studies from different regions have assessed this regimen in routine clinical practice and reported pCR rates ranging from 50.0% to 63.9% (12–16). This variability underscores the need for robust real-world data and reliable predictive tools to identify patients most likely to benefit from neoadjuvant immunotherapy. In parallel, machine learning (ML), an important subfield of artificial intelligence (AI), has increasingly been applied to predict treatment response and prognosis in cancer (17). Unlike conventional statistical methods, ML techniques are particularly effective at identifying complex patterns in large clinical datasets. They allow for the integration of multidimensional variables and the modeling of complex nonlinear relationships (18, 19).

Therefore, we conducted this real-world study to further evaluate the effectiveness of pembrolizumab in TNBC and to develop and validate machine learning–based models for predicting treatment response and prognosis.

## Methods

### Patients

A total of 248 TNBC patients who received neoadjuvant therapy followed by surgery were retrospectively enrolled at Sichuan Cancer Hospital between May 2023 and December 2025. Inclusion criteria were (1): female patients aged ≥18 years, (2) pathologically confirmed TNBC, defined according to the guidelines of the American Society of Clinical Oncology/College of American Pathologists (ASCO/CAP), (3) receipt of NACT with or without pembrolizumab, followed by definitive surgery, and (4) availability of relatively complete clinical data, case report forms, and treatment-related information. Exclusion criteria included: (1) presence of distant metastasis or concurrent malignant tumors, (2) severe comorbidities that could affect the efficacy or tolerability of neoadjuvant treatment (e.g., pre-existing autoimmune diseases, uncontrolled cardiovascular disease, active infection, severe hepatic/renal dysfunction, etc.), and (3) substantial missing clinical data.

This retrospective study was approved by the Ethics Committee of Sichuan Cancer Hospital (KY-2025-151) and complied with the standards of the Declaration of Helsinki. The Ethics Committee abandoned the informed consent form because it was a retrospective study.

### Neoadjuvant treatment and definitive surgery

Patients were categorized into two groups according to whether pembrolizumab was administered during neoadjuvant treatment: the pembrolizumab-chemotherapy group and the chemotherapy-alone group. Treatment strategies were determined by experienced physicians based on the Chinese Society of Clinical Oncology (CSCO) guidelines and patient preferences. Following completion of neoadjuvant therapy, all patients underwent definitive surgery.

### Response evaluation

The primary endpoint of this study was pCR. pCR was defined as the absence of residual invasive carcinoma in the resected breast specimen and all sampled regional lymph nodes after completion of neoadjuvant therapy (ypT0/Tis ypN0, allowing residual ductal carcinoma *in situ*). Breast MRI and ultrasound were used for radiological assessment. For RECIST 1.1 measurements, measurable solid lesions (≥10 mm) were measured by the longest diameter, and pathological lymph nodes (≥15 mm) by the short axis. The sum of longest diameters(SLD) of target lesions was calculated and used for response evaluation. Overall response was determined by changes in SLD for target lesions, qualitative changes in non-target lesions, and the presence of new lesions (CR: disappearance of all target lesions; PR: ≥30% decrease in SLD; PD: ≥20% increase in SLD or new/unequivocally progressive lesions; SD: <30% decrease and <20% increase in SLD).

### Machine learning models for pCR

Patients with incomplete baseline clinical variables or incomplete outcome information were excluded at the initial data-cleaning stage before development. All patients were randomly divided into two groups in a 7:3 ratio: the training cohort (n=174) and the validation cohort (n=74). The training cohort was used to identify predictive features, develop algorithms, and perform validation. Eight machine learning models were developed to predict pCR after neoadjuvant therapy, including decision tree (DT), elastic net (Enet), k-nearest neighbors (KNN), logistic regression (LR), multilayer perceptron (MLP), random forest (RF), regularized support vector machine (RSVM), and eXtreme Gradient Boosting (XGBoost), with 5-fold cross-validation used to robustly evaluate model performance.

In the validation cohort, model performance was assessed using receiver operating characteristic–area under the curve (ROC-AUC), accuracy, Cohen’s kappa, sensitivity, specificity, positive predictive value (PPV), negative predictive value (NPV), Matthews correlation coefficient (MCC), and balanced accuracy. Decision curve analysis (DCA) and calibration curves were further used to evaluate clinical utility and calibration.

SHAP values were used to assess the significance of features and understand their impact on model predictions.

### Statistical analysis

Categorical variables were processed using chi-square tests, while continuous variables were handled using t-tests for normally distributed data or Mann-Whitney U tests for non-normally distributed data. Propensity score matching (PSM) was used to eliminate baseline differences between the pembrolizumab-chemotherapy and chemotherapy-alone groups. Propensity scores were estimated using a logistic regression model based on baseline covariates, and patients in the two groups were then matched in a 1:3 ratio. All statistical analyses were conducted using R software. A p-value of <0.05 was considered statistically significant.

## Result

### Patient characteristics

Before PSM, there were significant differences between the pembrolizumab-chemotherapy (n = 40) and chemotherapy-alone group (n = 208) groups in terms of hemoglobin (HB) and platelets (PLT, all P < 0.05). After PSM, no significant differences in baseline characteristics were observed between the two groups (**Table 1**).

**Table 1:** Baseline characteristics of the patients before and after PSM.

Variable		Before PSM			After PSM		
	ALL	C	P+C	*p*-value	C	P+C	*p*-value
Patients	N=248	N=208	N=40		N=102	N=36	
Age (mean ± SD)	50.1 ± 11.4	50.7 ± 11.2	46.8 ± 11.8	0.059	48.0 ± 11.6	48.0 ± 11.3	0.972
Post-menopausal				0.404			0.939
No	100 (40.3%)	81 (38.9%)	19 (47.5%)		48 (47.1%)	16 (44.4%)	
Yes	148 (59.7%)	127 (61.1%)	21 (52.5%)		54 (52.9%)	20 (55.6%)	
Hypertension				1			1
No	203 (81.9%)	170 (81.7%)	33 (82.5%)		85 (83.3%)	30 (83.3%)	
Yes	45 (18.1%)	38 (18.3%)	7 (17.5%)		17 (16.7%)	6 (16.7%)	
BMI (mean ± SD)	23.9 ± 3.29	24.0 ± 3.35	23.3 ± 2.93	0.205	23.5 ± 2.86	23.5 ± 3.02	0.932
Location				0.363			0.993
Left	131 (52.8%)	113 (54.3%)	18 (45.0%)		49 (48.0%)	18 (50.0%)	
Right	117 (47.2%)	95 (45.7%)	22 (55.0%)		53 (52.0%)	18 (50.0%)	
Focality				0.544			1
Unifocal	192 (77.4%)	163 (78.4%)	29 (72.5%)		78 (76.5%)	27 (75.0%)	
Multifocal/multicentric	56 (22.6%)	45 (21.6%)	11 (27.5%)		24 (23.5%)	9 (25.0%)	
SLD (mean ± SD,cm)	3.96 ± 1.95	3.89 ± 1.92	4.28 ± 2.09	0.284	4.00 ± 2.02	4.21 ± 1.98	0.581
Ki67 (mean ± SD)	54.6 ± 23.5	54.2 ± 23.3	56.9 ± 24.7	0.532	54.3 ± 23.0	56.0 ± 25.2	0.732
HER2				0.622			0.977
0	99 (39.9%)	81 (38.9%)	18 (45.0%)		41 (40.2%)	15 (41.7%)	
1+	78 (31.5%)	65 (31.2%)	13 (32.5%)		36 (35.3%)	12 (33.3%)	
2+/F-	71 (28.6%)	62 (29.8%)	9 (22.5%)		25 (24.5%)	9 (25.0%)	
T				0.9			0.928
1	24 (9.68%)	20 (9.62%)	4 (10.0%)		11 (10.8%)	3 (8.33%)	
2	179 (72.2%)	150 (72.1%)	29 (72.5%)		75 (73.5%)	27 (75.0%)	
3	25 (10.1%)	22 (10.6%)	3 (7.50%)		10 (9.80%)	3 (8.33%)	
4	20 (8.06%)	16 (7.69%)	4 (10.0%)		6 (5.88%)	3 (8.33%)	
N				0.07			0.976
0	106 (42.7%)	91 (43.8%)	15 (37.5%)		38 (37.3%)	14 (38.9%)	
1	87 (35.1%)	70 (33.7%)	17 (42.5%)		44 (43.1%)	16 (44.4%)	
2	29 (11.7%)	28 (13.5%)	1 (2.50%)		6 (5.88%)	1 (2.78%)	
3	26 (10.5%)	19 (9.13%)	7 (17.5%)		14 (13.7%)	5 (13.9%)	
Stage				0.88			1
I	5 (2.02%)	5 (2.40%)	0 (0.00%)				
II	166 (66.9%)	138 (66.3%)	28 (70.0%)		74 (72.5%)	26 (72.2%)	
III	77 (31.0%)	65 (31.2%)	12 (30.0%)		28 (27.5%)	10 (27.8%)	
WBC (mean ± SD)	6.17 ± 2.01	6.09 ± 2.03	6.56 ± 1.89	0.16	6.54 ± 2.09	6.34 ± 1.85	0.597
NEU (mean ± SD)	4.14 ± 1.70	4.08 ± 1.72	4.44 ± 1.59	0.203	4.44 ± 1.69	4.23 ± 1.52	0.485
LYM (mean ± SD)	1.58 ± 0.51	1.56 ± 0.51	1.63 ± 0.52	0.443	1.63 ± 0.56	1.65 ± 0.54	0.874
HB (mean ± SD)	132 ± 14.3	131 ± 15.1	136 ± 8.28	0.006	137 ± 11.1	136 ± 8.23	0.759
PLT (mean ± SD)	219 ± 58.7	215 ± 58.2	243 ± 56.1	0.005	234 ± 57.2	235 ± 48.9	0.951
CRP (mean ± SD)	3.15 ± 8.73	2.85 ± 7.55	4.73 ± 13.3	0.39	3.38 ± 9.53	3.74 ± 12.4	0.875
ALB (mean ± SD)	44.1 ± 2.94	44.0 ± 2.92	44.6 ± 3.01	0.29	44.5 ± 2.68	44.4 ± 3.00	0.883
Cr (mean ± SD)	57.3 ± 9.19	57.3 ± 9.15	57.6 ± 9.50	0.821	57.1 ± 8.54	58.2 ± 9.76	0.532
LDH (mean ± SD)	191 ± 67.8	186 ± 54.9	215 ± 111	0.117	194 ± 70.9	201 ± 99.3	0.712
TC (mean ± SD)	5.01 ± 1.01	5.00 ± 1.01	5.06 ± 1.00	0.739	5.12 ± 1.08	5.12 ± 1.00	0.995

PSM, propensity score matching; C, the chemotherapy-alone group; P+C, the pembrolizumab-chemotherapy group; SLD, sum of diameters of target lesions according to RECIST version 1.1; HER2 2+/F-, HER2 2+ with FISH negative.

### pCR and radiological response

Both before and after PSM, the pembrolizumab–chemotherapy group achieved a higher pCR rate than the chemotherapy-alone group (before: 50.0% vs. 30.8%, p = 0.030; after: 47.2% vs. 26.5%, p = 0.037; **Table 2**).

**Table 2:** pCR^*^ and radiological response^†^ before and after PSM.

Variable		Before PSM			After PSM		
	ALL	C	P+C	p-value	C	P+C	p-value
Patients	N=248	N=208	N=40		N=102	N=36	
pCR	84 (33.9%)	64 (30.8%)	20 (50.0%)	0.03	27 (26.5%)	17 (47.2%)	0.037
Radiological response				0.262			0.124
CR	41 (16.5%)	31 (14.9%)	10 (25.0%)		11 (10.8%)	9 (25.0%)	
PR	151 (60.9%)	128 (61.5%)	23 (57.5%)		68 (66.7%)	21 (58.3%)	
SD	42 (16.9%)	38 (18.3%)	4 (10.0%)		20 (19.6%)	4 (11.1%)	
PD	14 (5.65%)	11 (5.29%)	3 (7.50%)		3 (2.94%)	2 (5.56%)	

*According to the current staging criteria of the American Joint Committee on Cancer and assessment by the local pathologist at the time of definitive surgery after completion of neoadjuvant systemic therapy, pCR was defined as the absence of residual invasive carcinoma in the resected breast specimen and all sampled regional lymph nodes after completion of neoadjuvant therapy (ypT0/Tis ypN0, allowing residual ductal carcinoma *in situ*).

†Radiological response to neoadjuvant therapy in breast cancer was assessed according to the Response Evaluation Criteria in Solid Tumors (RECIST), version 1.1.

In addition, before PSM, the rates of CR, PR, SD, and PD were 25.0%, 57.5%, 10.0%, and 7.5% in the pembrolizumab–chemotherapy group, respectively, compared with 14.9%, 61.5%, 18.3%, and 5.3% in the chemotherapy-alone group (**Table 2**).

### Selection of variables associated with pCR

LASSO regression selected six variables associated with pCR, including pembrolizumab, age, SLD, Ki67, N stage, and clinical stage (**Supplementary Figure 1**). Subsequently, univariate and multivariate logistic regression analyses showed that pembrolizumab, SLD, Ki67, and N stage were independent predictors of pCR (**Table 3**).

**Table 3:** Univariate and multivariate logistic regression analysis of pCR.

Variable	Univariate Logistic regression			Multivariate Logistic regression		
	OR	95%CI	p-value	OR	95%CI	p-value
Pembrolizumab (yes/no)	2.25	1.13-4.47	0.02	2.83	1.26-6.36	0.012
Age	0.95	0.93-0.98	<0.001	0.98	0.95-1.00	0.084
SLD	0.77	0.65-0.91	0.002	0.80	0.65-0.98	0.032
Ki67	1.03	1.02-1.04	<0.001	1.03	1.01-1.04	<0.001
N							
0	1.00					
1	0.43	0.23-0.78	0.006	0.49	0.25-0.96	0.038
2	0.29	0.11-0.78	0.013	0.46	0.09-2.44	0.364
3	0.20	0.07-0.63	0.006	0.28	0.05-1.68	0.165
Stage							
1	1.00					
2	0.17	0.02-1.51	0.111	0.34	0.03-3.61	0.371
3	0.06	0.01-0.54	0.012	0.33	0.02-5.60	0.441

SLD, sum of diameters of target lesions according to RECIST version 1.1.

### Machine learning

**Figure 1:**
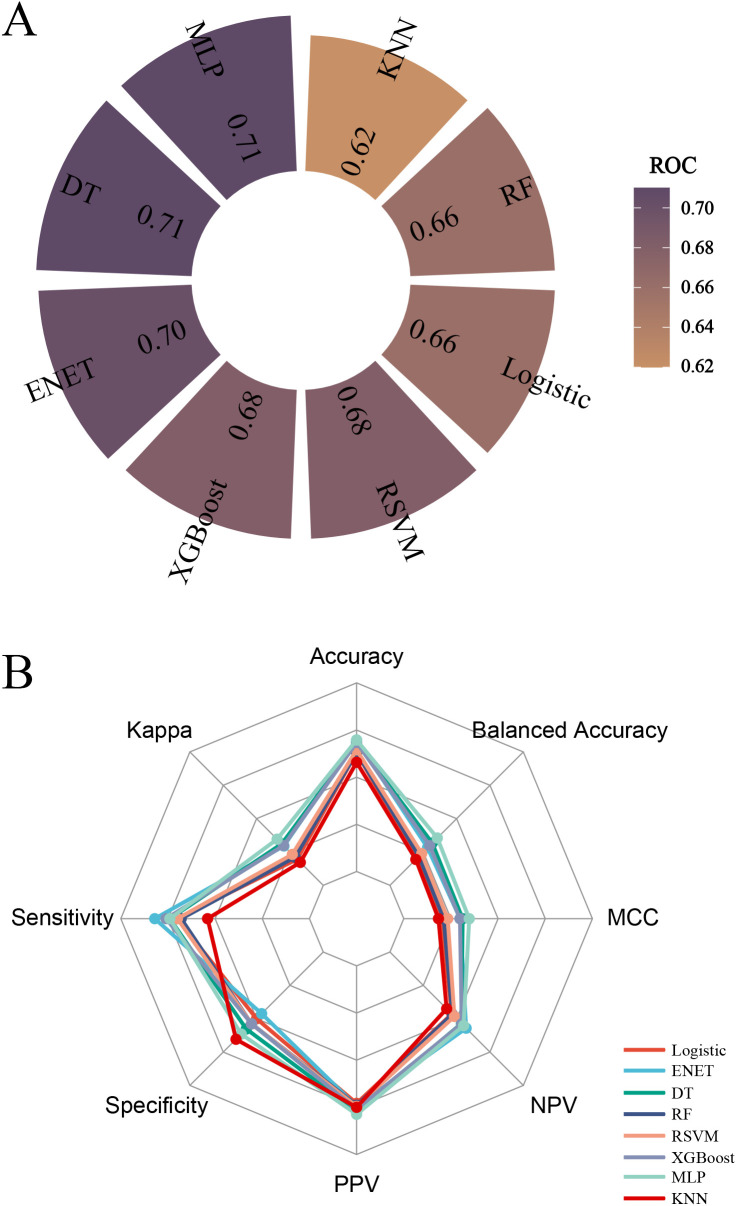
Performance evaluation of machine learning models. **(A)** Comparison of ROC–AUC values illustrating the discriminative ability of each model for predicting pCR; **(B)** Comparison of multiple performance metrics across eight machine learning models in the validation cohort.

**Figure 2:**
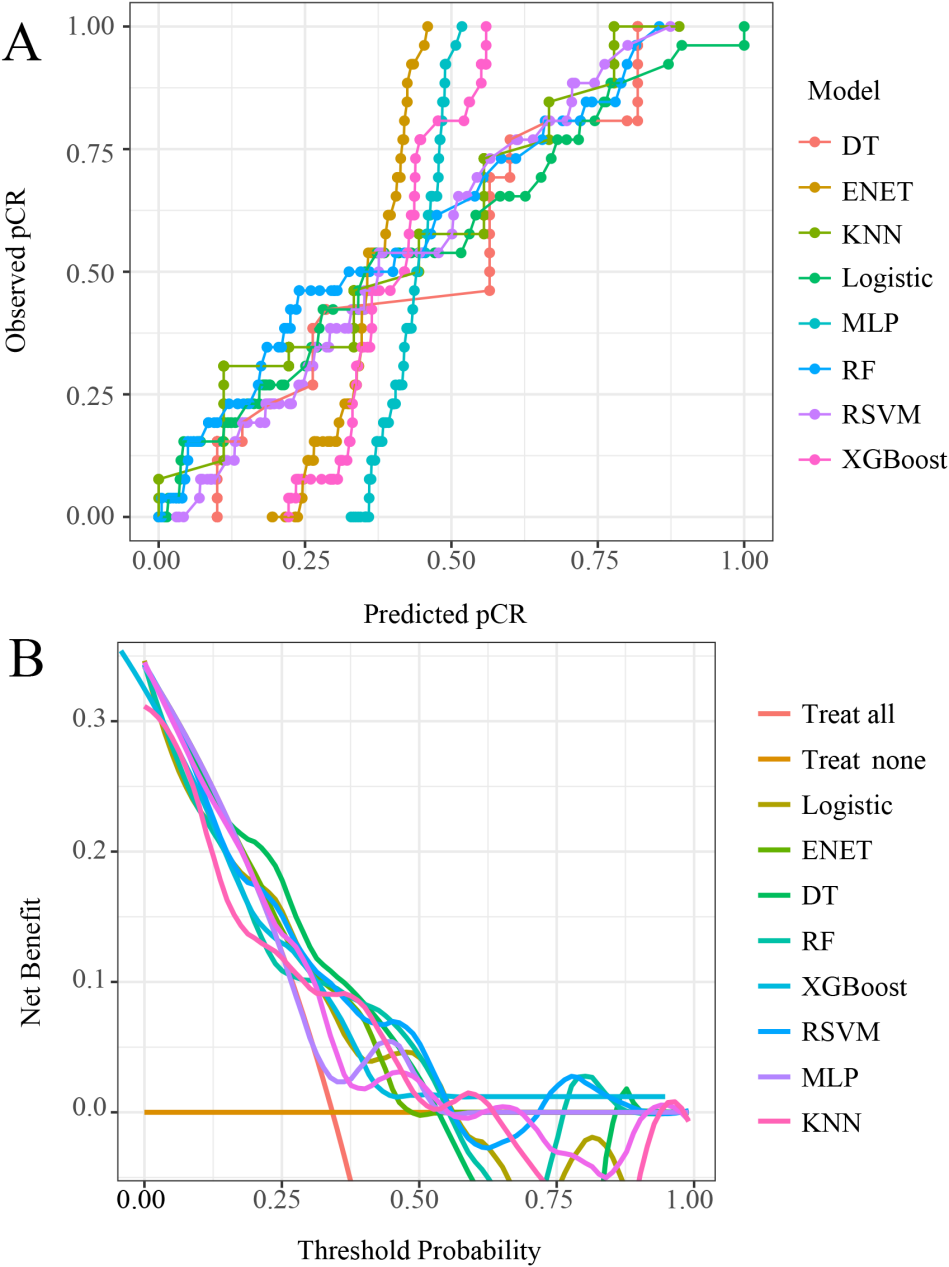
Model calibration and clinical utility in the validation cohort. **(A)** Calibration curves of eight machine learning models, showing the agreement between predicted probabilities and observed pCR outcomes; **(B)** Decision curve analysis of eight machine learning models across a range of threshold probabilities. “Treat all” and “treat none” indicate strategies in which all or no patients, respectively, would receive the intervention.

In the training cohort, pembrolizumab, age, SLD, Ki67, N stage, and clinical stage were incorporated into all eight machine learning models to predict pCR. In the validation cohort, both the MLP and DT models achieved the highest ROC-AUC of 0.71 (**Figure 1A**). Across other metrics, including accuracy, Cohen’s kappa, sensitivity, specificity, PPV, NPV, MCC, and balanced accuracy, the MLP model outperformed the DT model (**Figure 1B**). The calibration curve (**Figure 2A**) and DCA (**Figure 2B**) further indicated that the MLP model showed good calibration and clinical utility.

### SHAP explanation

The SHAP plot (**Supplementary Figure 2**) confirmed that Ki67 was the most critical factor influencing pCR, with higher values contributing positively to pCR prediction, while younger age and smaller SLD were associated with a higher likelihood of achieving pCR.

## Discussion

Studies have shown that achieving a pCR is associated with improved long-term outcomes in TNBC, making efforts to increase pCR rates and to predict pCR early clinically meaningful (7, 8). This study confirmed that adding neoadjuvant pembrolizumab was associated with a higher pCR rate than conventional chemotherapy alone, and our ML models showed good performance in predicting pCR, offering a potential tool to identify patients who may benefit from neoadjuvant pembrolizumab-chemotherapy therapy (20–23).

The KEYNOTE-522 trial established pembrolizumab plus neoadjuvant chemotherapy as a new standard of care by demonstrating significant improvements in pCR, EFS, and overall survival in TNBC (10, 11, 24). Subsequent real-world studies from different regions have reported pCR rates broadly comparable to those observed in KEYNOTE-522, while also underscoring greater patient heterogeneity, lower treatment completion rates, and increased complexity of toxicity management in routine clinical practice (12, 13, 25, 26). Consistent with these findings, our study showed that the pembrolizumab-chemotherapy group maintained a higher pCR rate both before and after PSM in the real-world setting, solidifying clinicians’ confidence to use this regimen.

In the machine learning analysis, the predictive model achieved moderate discriminative performance in the validation cohort (AUC = 0.71). Compared with traditional models, the machine learning model showed better predictive performance. For patients with a low predicted probability of pCR before treatment, more intensive therapy and closer response monitoring may be considered. The SHAP plot identified Ki-67 as one of the most influential predictors of pCR. Consistent with prior studies, higher Ki-67 levels have been linked to higher pCR rates in breast cancer (27, 28). As a marker of tumor proliferative activity, elevated Ki-67 expression is generally associated with increased sensitivity to neoadjuvant treatments that target rapidly dividing cells, supporting the biological plausibility of our findings (29, 30). In addition, SLD and nodal status contributed substantially to model performance, consistent with established evidence linking tumor burden to treatment response. These findings may help clinicians rapidly determine the probability of neoadjuvant treatment benefit for TNBC patients.

Notably, many pCR prediction models have been built on imaging-derived features such as radiomics or deep learning analyses of MRI and digital pathology (31). Although these approaches can achieve strong discrimination in selected cohorts, they often rely on highly standardized acquisition and processing pipelines. Variations in scanners, acquisition parameters, preprocessing, segmentation, and inter-institutional protocols may introduce substantial bias and reduce reproducibility and generalizability in routine practice (32–34). In contrast, our model relies on routinely collected clinical variables, which supports broader clinical implementation.

Although no significant differences were observed in the imaging responses based on the RECIST version 1.1 between treatment groups, this result can be reasonably explained. Size-based imaging criteria primarily capture changes in tumor dimensions and may underestimate therapeutic efficacy when immune-mediated mechanisms are involved, as immune cell infiltration, stromal remodeling, and tumor necrosis do not necessarily translate into immediate tumor shrinkage on imaging (35, 36). In contrast, pathological assessment directly quantifies residual viable tumor burden and has been consistently shown to better reflect treatment-induced tumor eradication.

Nevertheless, our study has some limitations. First, heterogeneity in chemotherapy regimens in real-world practice may induce confounding, and the non-random use of immunotherapy may also introduce selection bias. Second, its retrospective single-center design introduces potential residual confounding despite statistical adjustment, and the lack of long-term follow-up precluded direct assessment of whether improved pCR translates into survival benefit. Third, the predictive model has not yet undergone external validation, and its generalizability requires confirmation in independent multi-center cohorts. Future work should include larger multi-center cohorts with longer follow-up to improve generalizability and evaluate survival outcomes. Prospective studies, clearer treatment reporting, and external validation are needed to reduce confounding. In addition, model performance should be further optimized in terms of calibration and clinical predictive accuracy to support clinical use.

## Conclusion

In conclusion, in real-world practice, neoadjuvant pembrolizumab combined with chemotherapy further improved pCR rates in TNBC, and the MLP-based model demonstrated good performance for predicting pCR.

## Data Availability

The raw data supporting the conclusions of this article will be made available by the authors, without undue reservation.

## References

[1] Bianchini G De Angelis C Licata L Gianni L . Treatment landscape of triple-negative breast cancer - expanded options, evolving needs. Nat Rev Clin Oncol. (2022) 19:91–113. doi: 10.1038/s41571-021-00565-2. PMID: 34754128

[2] Gianni L Huang CS Egle D Bermejo B Zamagni C Thill M . Pathologic complete response (pCR) to neoadjuvant treatment with or without atezolizumab in triple-negative, early high-risk and locally advanced breast cancer: NeoTRIP Michelangelo randomized study. Ann Oncol. (2022) 33:534–43. doi: 10.1016/j.annonc.2022.02.004. PMID: 35182721

[3] Foulkes WD Smith IE Reis-Filho JS . Triple-negative breast cancer. N Engl J Med. (2010) 363:1938–48. doi: 10.1056/NEJMra1001389. PMID: 21067385

[4] Giaquinto AN Sung H Newman LA Freedman RA Smith RA Star J . Breast cancer statistics 2024. CA Cancer J Clin. (2024) 74:477–95. doi: 10.3322/caac.21863. PMID: 39352042

[5] Gradishar WJ Moran MS Abraham J Abramson V Aft R Agnese D . NCCN guidelines® Insights: breast cancer, version 5.2025. J Natl Compr Canc Netw. (2025) 23:426–36. doi: 10.6004/jnccn.2025.0053. PMID: 41213254

[6] Bianchini G Balko JM Mayer IA Sanders ME Gianni L . Triple-negative breast cancer: challenges and opportunities of a heterogeneous disease. Nat Rev Clin Oncol. (2016) 13:674–90. doi: 10.1038/nrclinonc.2016.66. PMID: 27184417 PMC5461122

[7] Cortazar P Zhang L Untch M Mehta K Costantino JP Wolmark N . Pathological complete response and long-term clinical benefit in breast cancer: the CTNeoBC pooled analysis. Lancet. (2014) 384:164–72. doi: 10.1016/S0140-6736(13)62422-8. PMID: 24529560

[8] Conforti F Pala L Sala I Oriecuia C De Pas T Specchia C . Evaluation of pathological complete response as surrogate endpoint in neoadjuvant randomized clinical trials of early stage breast cancer: systematic review and meta-analysis. BMJ. (2021) 375:e066381. doi: 10.1136/bmj-2021-066381. PMID: 34933868 PMC8689398

[9] Huang M O'Shaughnessy J Zhao J Haiderali A Cortés J Ramsey SD . Association of pathologic complete response with long-term survival outcomes in triple-negative breast cancer: a meta-analysis. Cancer Res. (2020) 80:5427–34. doi: 10.1158/0008-5472.CAN-20-1792. PMID: 32928917

[10] Schmid P Cortes J Pusztai L McArthur H Kümmel S Bergh J . Pembrolizumab for early triple-negative breast cancer. N Engl J Med. (2020) 382:810–21. doi: 10.1056/NEJMoa1910549. PMID: 32101663

[11] Schmid P Cortes J Dent R Pusztai L McArthur H Kümmel S . Event-free survival with pembrolizumab in early triple-negative breast cancer. N Engl J Med. (2022) 386:556–67. doi: 10.1056/NEJMoa2112651. PMID: 35139274

[12] Karci E Bilici A Bayram B Celayir M Ozyurt N Uluc BO . Neoadjuvant pembrolizumab plus chemotherapy in early-stage triple-negative breast cancer: a nationwide retrospective Turkish Oncology Group study. Cancers (Basel). (2024) 16:3389. doi: 10.3390/cancers16193389. PMID: 39410009 PMC11475936

[13] Marhold M Udovica S Halstead A Hirdler M Ferner M Wimmer K . Emergence of immune-related adverse events correlates with pathological complete response in patients receiving pembrolizumab for early triple-negative breast cancer. Oncoimmunology. (2023) 12:2275846. doi: 10.1080/2162402X.2023.2275846. PMID: 38025838 PMC10653620

[14] Connors C Valente SA ElSherif A Escobar P Chichura A Kopicky L . Real-world outcomes with the KEYNOTE-522 regimen in early-stage triple-negative breast cancer. Ann Surg Oncol. (2025) 32:912–21. doi: 10.1245/s10434-024-16390-7. PMID: 39436619 PMC11843215

[15] Aoyama Y Ozaki Y Kizawa R Masuda J Kawai S Kurata M . Efficacy and feasibility of neoadjuvant pembrolizumab plus chemotherapy for early-stage triple-negative and estrogen receptor low, HER2-negative breast cancer: a Japanese single-institution real-world study. Breast Cancer. (2025) 32:329–36. doi: 10.1007/s12282-024-01657-4. PMID: 39644440

[16] LeVee A Wong M Flores S Ruel N McArthur H Waisman J . Impact of neoadjuvant pembrolizumab adherence on pathologic complete response in triple-negative breast cancer: a real-world analysis. Oncologist. (2024) 29:566–74. doi: 10.1093/oncolo/oyae064. PMID: 38656345 PMC11224989

[17] Eloranta S Boman M . Predictive models for clinical decision making: deep dives in practical machine learning. J Intern Med. (2022) 292:278–95. doi: 10.1111/joim.13483. PMID: 35426190 PMC9544754

[18] Obermeyer Z Emanuel EJ . Predicting the future - big data, machine learning, and clinical medicine. N Engl J Med. (2016) 375:1216–9. doi: 10.1056/NEJMp1606181. PMID: 27682033 PMC5070532

[19] Sammut SJ Crispin-Ortuzar M Chin SF Provenzano E Bardwell HA Ma W . Multi-omic machine learning predictor of breast cancer therapy response. Nature. (2022) 601:623–9. doi: 10.1038/s41586-021-04278-5. PMID: 34875674 PMC8791834

[20] Ogier du Terrail J Leopold A Joly C Béguier C Andreux M Maussion C . Federated learning for predicting histological response to neoadjuvant chemotherapy in triple-negative breast cancer. Nat Med. (2023) 29:135–46. doi: 10.1038/s41591-022-02155-w. PMID: 36658418

[21] Wang S Song Y Ding J Li M Wang Y Bai Y . Development and validation of a new immune-inflammatory-nutritional score to predict pathological complete response in triple-negative breast cancer undergoing neoadjuvant chemotherapy: a two-center study. J Inflammation Res. (2025) 18:9365–78. doi: 10.2147/JIR.S526429. PMID: 40687145 PMC12276740

[22] Lee JS Yost SE Yuan Y . Neoadjuvant treatment for triple negative breast cancer: recent progresses and challenges. Cancers (Basel). (2020) 12:1404. doi: 10.3390/cancers12061404. PMID: 32486021 PMC7352772

[23] Luo L Wu M Li M Xin Y Wang Q Vardhanabhuti V . A large model for non-invasive and personalized management of breast cancer from multiparametric MRI. Nat Commun. (2025) 16:3647. doi: 10.1038/s41467-025-58798-z. PMID: 40246826 PMC12006510

[24] Schmid P Cortes J Dent R McArthur H Pusztai L Kümmel S . KEYNOTE-522 Investigators. Overall survival with pembrolizumab in early-stage triple-negative breast cancer. N Engl J Med. (2024) 391:1981–91. doi: 10.1056/NEJMoa2409932. PMID: 39282906

[25] Andrade MO Gutierres IG Tavares MC De Sousa IM Balint FC Marin Comini AC . Immune-related adverse events among patients with early-stage triple-negative breast cancer treated with pembrolizumab plus chemotherapy: real-world data from the Neo-Real/GBECAM 0123 study. Breast. (2025) 83:104473. doi: 10.1016/j.breast.2025.104473. PMID: 40240201 PMC12496205

[26] Giugliano F Valenza C Tarantino P Curigliano G . Immunotherapy for triple negative breast cancer: how can pathologic responses to experimental drugs in early-stage disease be enhanced? Expert Opin Investig Drugs. (2022) 31:855–74. doi: 10.1080/13543784.2022.2095260. PMID: 35762248

[27] Wang W Wu J Zhang P Fei X Zong Y Chen X . Prognostic and predictive value of Ki-67 in triple-negative breast cancer. Oncotarget. (2016) 7:31079–87. doi: 10.18632/oncotarget.9075. PMID: 27145269 PMC5058740

[28] Srivastava P Wang T Clark BZ Yu J Fine JL Villatoro TM . Clinical-pathologic characteristics and response to neoadjuvant chemotherapy in triple-negative low Ki-67 proliferation (TNLP) breast cancers. NPJ Breast Cancer. (2022) 8:51. doi: 10.1038/s41523-022-00415-z. PMID: 35444182 PMC9021249

[29] De Azambuja E Cardoso F de Castro GJ Colozza M Mano MS Durbecq V . Ki-67 as prognostic marker in early breast cancer: a meta-analysis of published studies involving 12,155 patients. Br J Cancer. (2007) 96:1504–13. doi: 10.1038/sj.bjc.6603756. PMID: 17453008 PMC2359936

[30] Häberle L Erber R Gass P Hein A Niklos M Volz B . Prediction of pathological complete response after neoadjuvant chemotherapy for HER2-negative breast cancer patients with routine immunohistochemical markers. Breast Cancer Res. (2025) 27:13. doi: 10.1186/s13058-025-01960-8. PMID: 39856787 PMC11759445

[31] Corredor G Bharadwaj S Pathak T Viswanathan VS Toro P Madabhushi A . A review of AI-based radiomics and computational pathology approaches in triple-negative breast cancer: current applications and perspectives. Clin Breast Cancer. (2023) 23:800–12. doi: 10.1016/j.clbc.2023.06.004. PMID: 37380569 PMC10733554

[32] Trebeschi S Drago SG Birkbak NJ Kurilova I Cǎlin AM Delli Pizzi A . Predicting response to cancer immunotherapy using noninvasive radiomic biomarkers. Ann Oncol. (2019) 30:998–1004. doi: 10.1093/annonc/mdz108. PMID: 30895304 PMC6594459

[33] Zhao X Bai JW Guo Q Ren K Zhang GJ . Clinical applications of deep learning in breast MRI. Biochim Biophys Acta Rev Cancer. (2023) 1878:188864. doi: 10.1016/j.bbcan.2023.188864. PMID: 36822377

[34] Tang W Jin C Kong Q Liu C Chen S Ding S . Development and validation of an MRI spatiotemporal interaction model for early noninvasive prediction of neoadjuvant chemotherapy response in breast cancer: a multicenter study. EClinicalMedicine. (2025) 85:103298. doi: 10.1016/j.eclinm.2025.103298. PMID: 40584836 PMC12205660

[35] Eisenhauer EA Therasse P Bogaerts J Schwartz LH Sargent D Ford R . New response evaluation criteria in solid tumors: revised RECIST guideline (version 1.1). Eur J Cancer. (2009) 45:228–47. doi: 10.1016/j.ejca.2008.10.026. PMID: 19097774

[36] Forde PM Chaft JE Smith KN Anagnostou V Cottrell TR Hellmann MD . Neoadjuvant PD-1 blockade in resectable lung cancer. N Engl J Med. (2018) 378:1976–86. doi: 10.1056/NEJMoa1716078. PMID: 29658848 PMC6223617

