# Supplementary material for: The role of AI-driven decision support in immunotherapy for predicting pathological complete response in triple-negative breast cancer
Source: Front Oncol. 2026 Apr 13;16:1798015. doi: 10.3389/fonc.2026.1798015 (PMC13111214; doi:10.3389/fonc.2026.1798015)

**Supplementary Fig. 1 LASSO feature selection.**
(A) Coefficient paths versus log(λ).
(B) 10-fold cross-validation curve (binomial deviance). Dotted lines indicate λ_min and λ_1se.


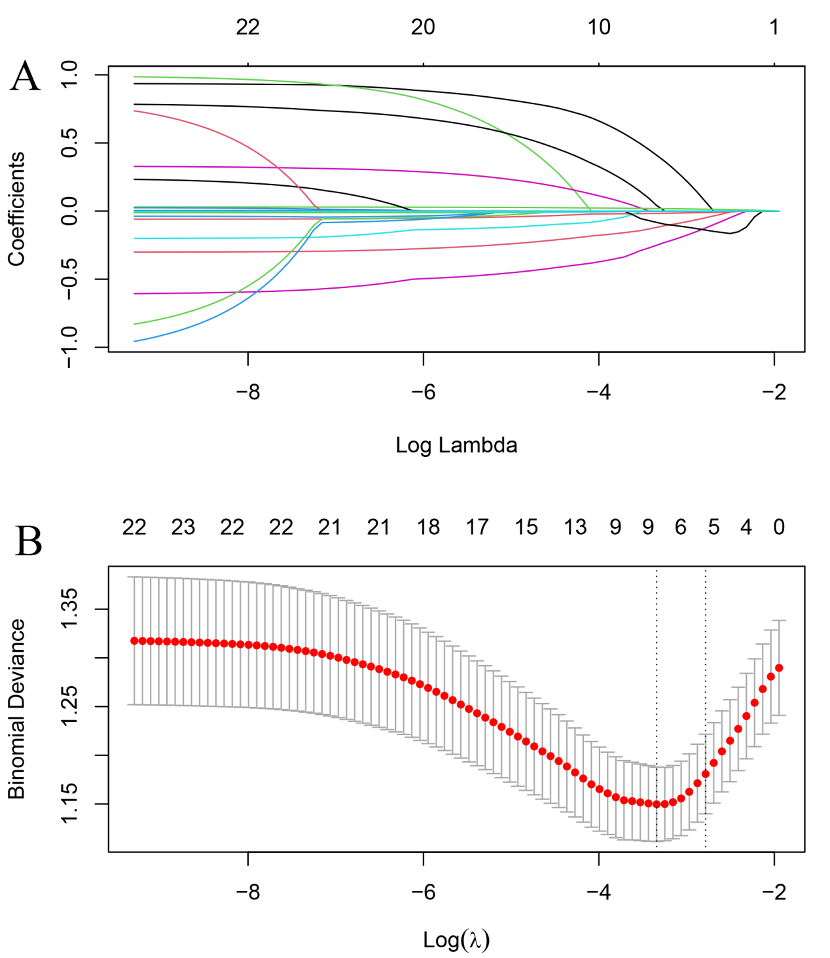


**Supplementary Fig. 2 SHAP summary plot for pCR prediction.**

SHAP summary plot showing the contribution of individual features to the prediction of pCR. Each dot represents a patient, with color indicating the feature value and the SHAP value reflecting the direction and magnitude of its influence on the model prediction.


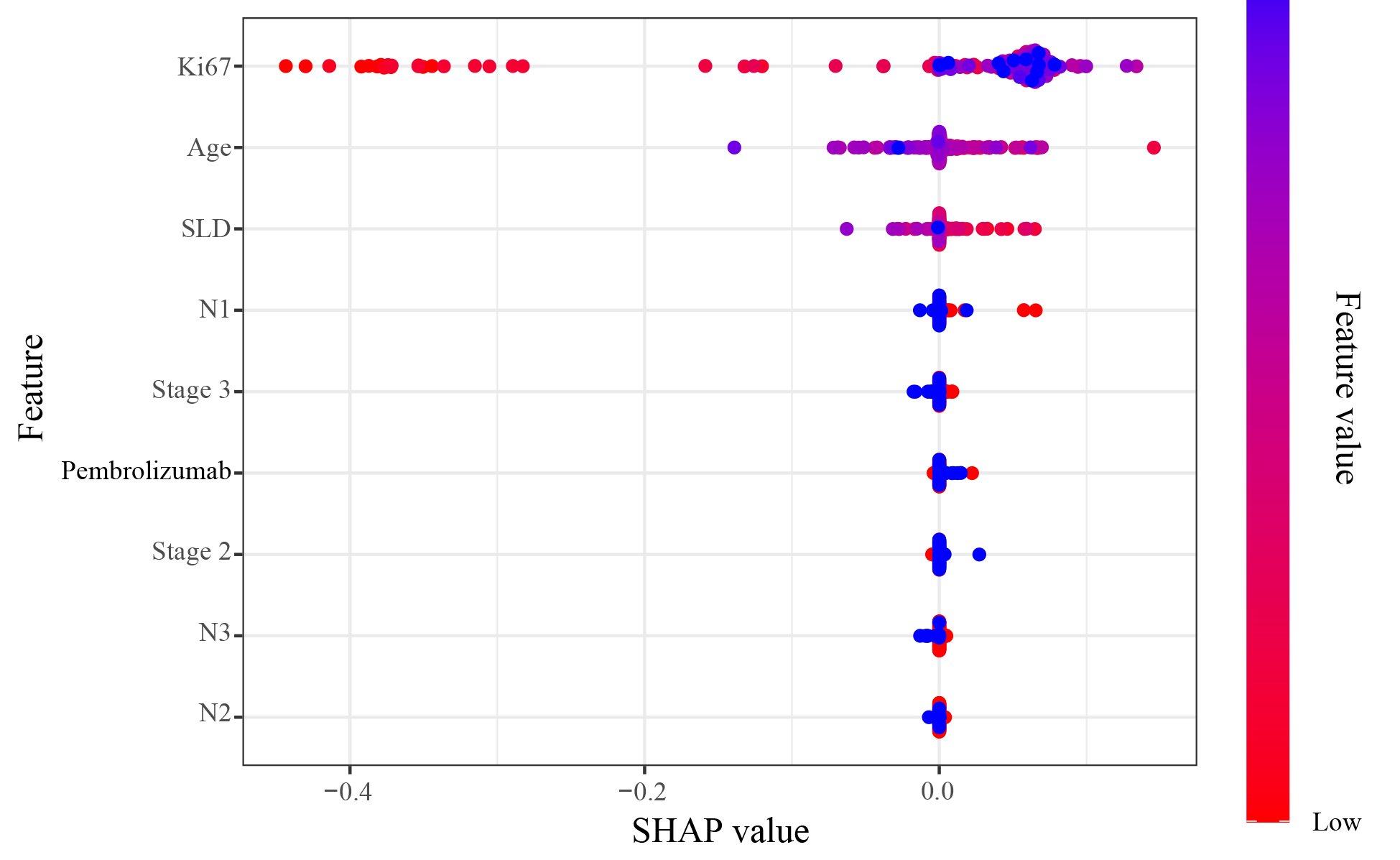

Supplement: Supplementary file 1 [file DataSheet1.docx]
